# Malic Enzyme 1 as a Novel Anti‐Ferroptotic Regulator in Hepatic Ischemia/Reperfusion Injury

**DOI:** 10.1002/advs.202205436

**Published:** 2023-02-25

**Authors:** Xuexian Fang, Jiawei Zhang, You Li, Yijing Song, Yingying Yu, Zhaoxian Cai, Fuzhi Lian, Jun Yang, Junxia Min, Fudi Wang

**Affiliations:** ^1^ Department of Nutrition and Toxicology Key Laboratory of Elemene Class Anti‐Cancer Chinese Medicines of Zhejiang Province School of Public Health Hangzhou Normal University Hangzhou 311121 China; ^2^ The Second Affiliated Hospital The First Affiliated Hospital School of Public Health Institute of Translational Medicine State Key Laboratory of Experimental Hematology Zhejiang University School of Medicine Hangzhou 310058 China; ^3^ The First Affiliated Hospital Basic Medical Sciences, School of Public Health Hengyang Medical School University of South China Hengyang 421001 China

**Keywords:** ferroptosis, hepatic ischemia/reperfusion injury, malate, malic enzyme 1, nicotinamide adenine dinucleotide phosphate

## Abstract

Ferroptosis has been linked to the pathogenesis of hepatic injury induced by ischemia/reperfusion (I/R). However, the mechanistic basis remains unclear. In this study, by using a mouse model of hepatic I/R injury, it is observed that glutathione (GSH) and cysteine depletion are associated with deficiency of the reducing power of nicotinamide adenine dinucleotide phosphate (NADPH). Genes involved in maintaining NADPH homeostasis are screened, and it is identified that I/R‐induced hepatic ferroptosis is significantly associated with reduced expression and activity of NADP^+^‐dependent malic enzyme 1 (Me1). Mice with hepatocyte‐specific Me1 gene deletion exhibit aggravated ferroptosis and liver injury under I/R treatment; while supplementation with L‐malate, the substrate of ME1, restores NADPH and GSH levels and eventually inhibits I/R‐induced hepatic ferroptosis and injury. A mechanistic study further reveals that downregulation of hepatic Me1 expression is largely mediated by the phosphatase and tensin homologue (PTEN)‐dependent suppression of the mechanistic target of rapamycin/sterol regulatory element‐binding protein 1 (mTOR/SREBP1) signaling pathway in hepatic I/R model. Finally, PTEN inhibitor, mTOR activator, or SREBP1 over‐expression all increase hepatic NADPH, block ferroptosis, and protect liver against I/R injury. Taken together, the findings suggest that targeting ME1 may provide new therapeutic opportunities for I/R injury and other ferroptosis‐related hepatic conditions.

## Graphical abstract

Hepatic ischemia/reperfusion (I/R) results in down‐regulation of Malic enzyme 1 (Me1), which is known to replenish the intracellular reducing equivalent and to maintain redox homeostasis. Loss of Me1 significantly increases the susceptibility to hepatic ferroptosis in mice, leading to aggravated I/R injury. This work highlights the important role of Me1 in protecting against ferroptosis‐mediated liver injury.

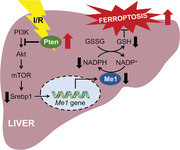

## 1 Introduction

Hepatic ischemia/reperfusion (I/R) injury is a major type of liver damages that frequently occurs in a series of clinical conditions, including liver resection, liver transplantation, and trauma surgery.^[^ ^1^ ^]^ As one of the main causes of early organ dysfunction and/or surgical failure, it has a significant impact on the patients' prognosis and rate of recovery.^[^ ^2^ ^]^ Thus, investigating the molecular mechanism underlying hepatic I/R injury is urgently needed to reveal potential targets for developing therapeutic strategies. However, highly intricate network of events that culminate in liver after I/R treatment has been discussed for decades and still remains unclear, resulting in an unresolved problem in the clinic.

Ferroptosis is a non‐apoptotic form of regulated cell death and has been implicated in the pathogenesis of a growing list of human diseases.^[^ ^3^, ^4^, ^5^ ^]^ Recent studies have suggested that ferroptosis may contribute a lot to hepatic I/R injury.^[^ ^6^ ^]^ Blocking ferroptosis by specific inhibitor, iron chelator, or antioxidant could significantly attenuate tissue injury and improve liver function after I/R treatment.^[^ ^7^, ^8^ ^]^ Although several ferroptosis‐associated genes and pathways have been implicated in liver diseases, the precise mechanism by which I/R treatment triggers ferroptosis is poorly understood.

Ferroptosis is driven by excessive accumulation of lipid hydroperoxides. And the cyst(e)ine/glutathione (GSH)/glutathione peroxidase 4 (GPX4) axis is the canonical and most frequently targeted pathway for suppressing ferroptosis. Specifically, the cystine/glutamate antiporter SLC7A11 imports cystine, which is further reduced to cysteine and the latter is used to synthesize GSH, a necessary cofactor for GPX4‐mediated defense system against lipid peroxidation.^[^ ^3^ ^]^ Nicotinamide adenine dinucleotide phosphate (NADPH) is the essential cofactor of reactions regulating either the reducing of cystine or recycling of reduced/oxidized glutathione (GSH/GSSG).^[^ ^9^ ^]^ Additionally, decreased NADPH abundance could serve as a biomarker for detecting hepatic ferroptosis.^[^ ^10^, ^11^ ^]^

Here, we systematically investigated the role of NADPH homeostasis in I/R‐induced hepatic ferroptosis, and identified cytosolic NADPH provider malic enzyme 1 (ME1) as a novel ferroptosis suppressor in the liver. Specifically deleting Me1 in mouse hepatocytes resulted in enhanced susceptibility to ferroptosis and exacerbated liver injury after I/R procedure. Conversely, supplementation of L‐malate, the substrate of ME1, increased NADPH abundance to shield the liver from ferroptosis and tissue damage. Together, our findings demonstrate that ME1 is a potential therapeutic target for treating hepatic I/R injury or other ferroptosis‐related disorders.

## 2 Results

### 2.1 NADPH Deficiency Drives Ferroptosis in Hepatic I/R Injury

**Figure 1:**
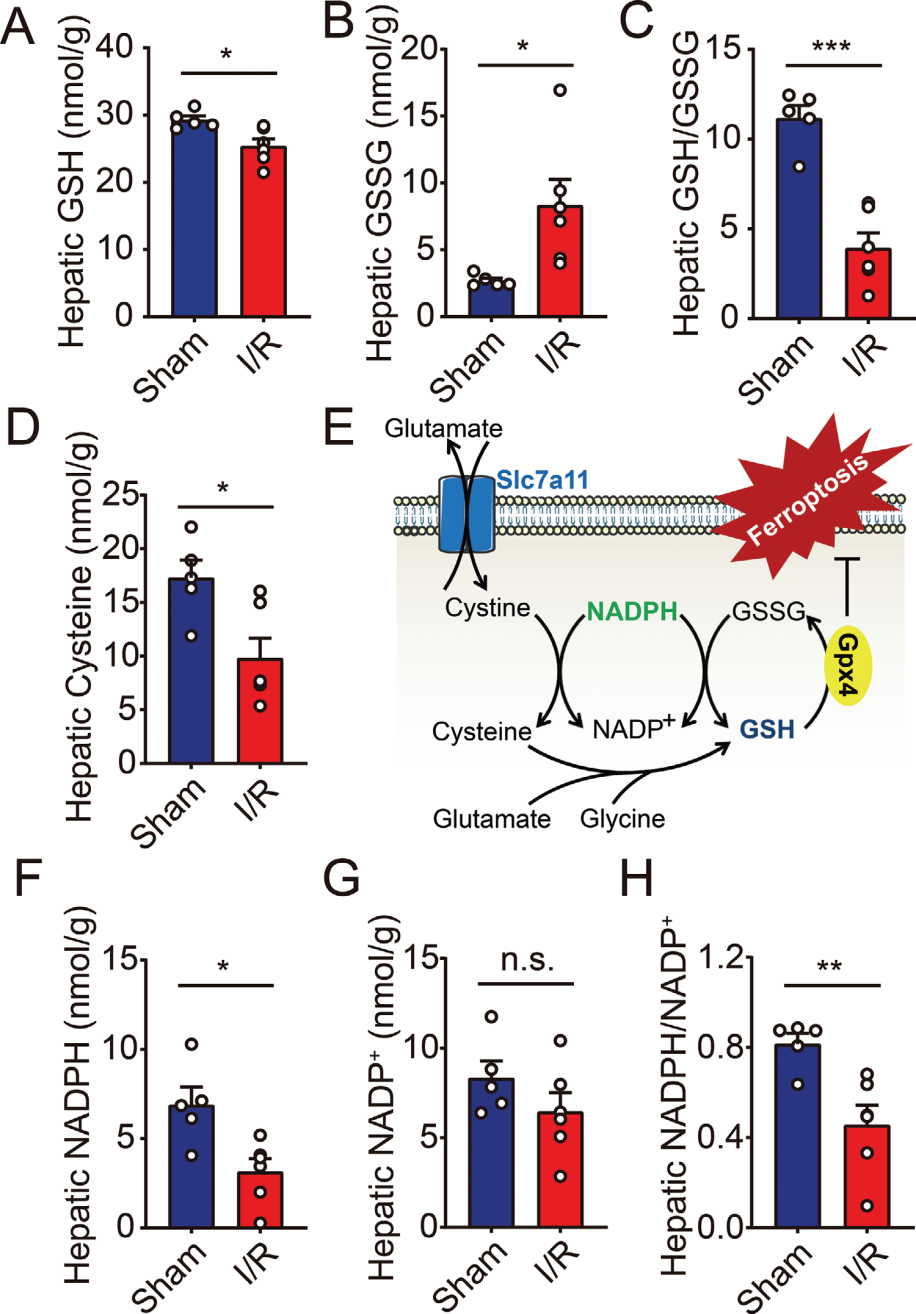
Hepatic I/R injury induces deficiency of either GSH or NADPH in mice. A–C) Hepatic levels of GSH (A), GSSG (B), and GSH/GSSG ratio (C) were measured in mice with sham or I/R injury. *n* = 5, 6. D) Hepatic levels of cysteine were measured in mice with sham or I/R injury. *n* = 5, 6. E) Schematic diagram depicting the key steps in the regulation of GSH biosynthesis and ferroptosis. F–H) Hepatic levels of NADPH (F), NADP^+^ (G), and NADPH/NADP^+^ ratio (H) were measured in mice with sham or I/R injury. *n* = 5, 6. Significance was calculated by Student's *t*‐test; *p < 0.05, **p < 0.01, ***p < 0.001, n.s. = not significant.

As a well‐established model to induce in vivo ferroptosis,^[^ ^7^ ^]^ hepatic I/R injury demonstrated important biochemical signatures of ferroptosis. In the liver subjected to I/R, GSH was depleted while GSSG was deposited, resulting in significantly reduced ratio of GSH/GSSG (**Figure** **1**A–C). GSH is a tripeptide composed of glutamate, cysteine, and glycine, but cysteine is assumed to be the rate‐limiting metabolite for the biosynthesis. Thus, we measured the hepatic contents of cysteine and observed similar depletion in the model of I/R injury (Figure 1D). As is well known, GPX4 utilizes GSH as a reducing agent to prevent ferroptosis, through which GSH is oxidized to GSSG. Then GSSG can be recycled back to GSH in the presence of NADPH. It is no coincidence that the reduction reaction converting cystine to cysteine requires NADPH too (Figure 1E). Therefore, we hypothesized that NADPH homeostasis could play a critical role in regulating hepatic ferroptosis during I/R operation via alteration of de novo synthesis as well as redox status of GSH. We further compared NADPH abundance in livers with or without I/R treatment, and found both depletion of hepatic NADPH and decrease in NADPH/NADP^+^ ratios (Figure 1F–H).

### 2.2 I/R Suppresses Hepatic Me1 Expression and Activity

**Figure 2:**
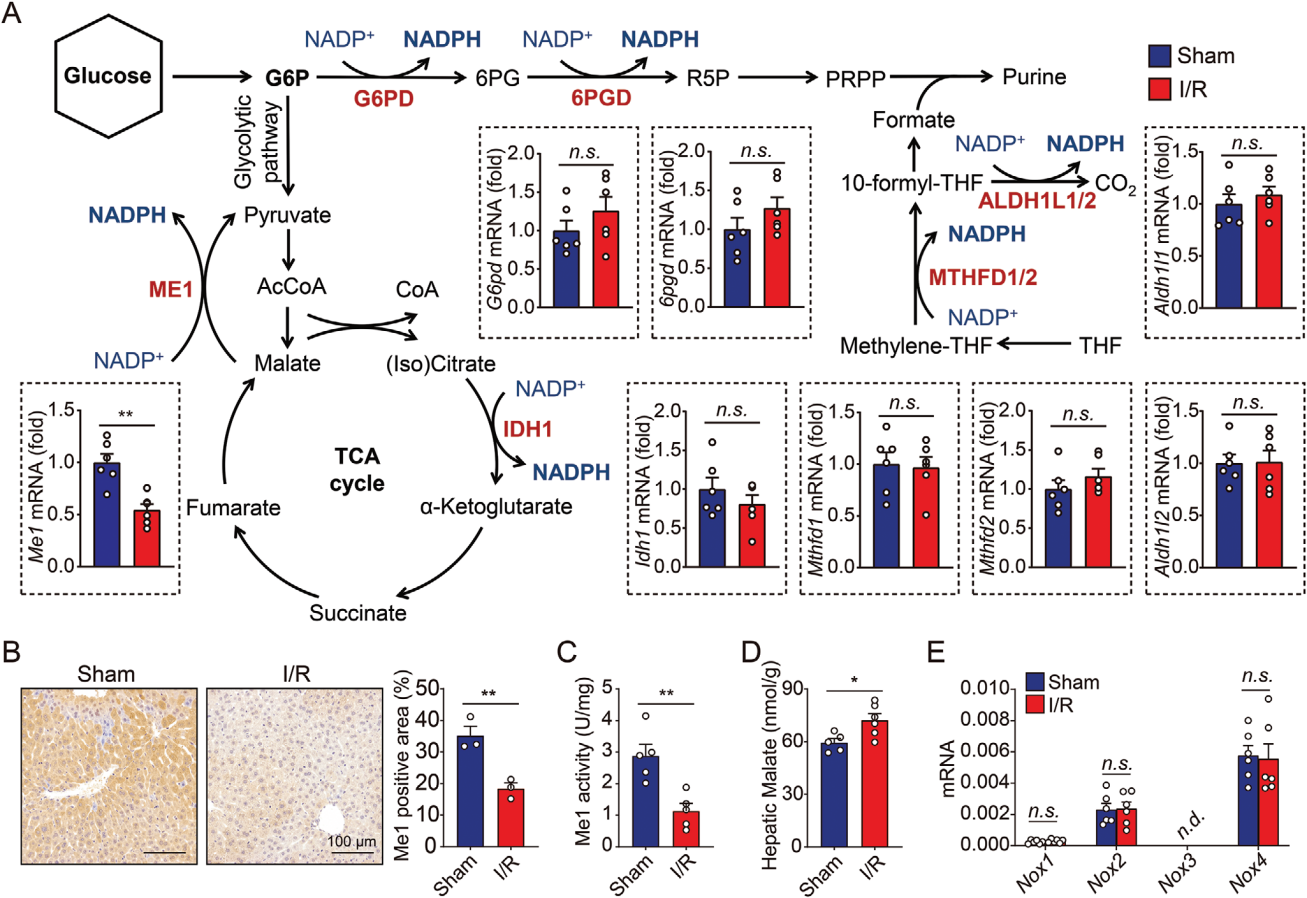
Me1 is suppressed in hepatic I/R injury. A) Targeted genes regulating NADPH production were analyzed in the livers of mice with sham or I/R injury. *n* = 6 per group. B) Representative immunohistochemistry images (left) and quantitative data (right) of Me1‐stained liver sections from mice with sham or I/R injury. C) Me1 activity was measured in liver homogenates obtained from mice with sham or I/R injury. *n* = 5 per group. D) Hepatic levels of malate were measured in mice with sham or I/R injury. *n* = 5, 6. E) Hepatic *Nox1*, *Nox2*, *Nox3*, and *Nox4* mRNA of were measured in mice with sham or I/R injury. *n* = 6 per group. Significance was calculated by Student's *t*‐test; *p < 0.05, **p < 0.01, ***p < 0.001, n.s. = not significant, n.d. = not detected.

On the basis of the above findings, we then designed a strategy to generally survey genes involved in NADPH metabolism. Previous studies have focused mainly on the pentose phosphate pathway (PPP) as the major contributor of cytosolic NADPH. In the past decade, emerging evidence has shifted this paradigm and demonstrated the involvement of alternative routes in cellular NADPH production.^[^ ^12^ ^]^ Among these NADPH‐generating enzymes, only *Me1* expression was remarkably downregulated in the liver by surgical treatment (**Figure** **2**A and Figure S1, Supporting Information). ME1 is responsible for oxidative decarboxylation of malate to pyruvate, concomitantly reducing NADP^+^ to NADPH.^[^ ^13^ ^]^ In consistence with the mRNA level, the protein expression of Me1 was markedly decreased in I/R‐treated livers (Figure 2B). In addition, we also found that I/R suppressed Me1 activity and caused slight accumulation of malate in the liver (Figure 2C,D). Finally, hepatic expressions of NADPH oxidases (NOXs), which consume NADPH to produce reactive oxygen species (ROS),^[^ ^14^ ^]^ were also measured. Results showed that *Nox1*, *Nox2*, and *Nox4* were detectable in the liver, but no difference was observed between sham and I/R groups, suggesting that NOXs are not responsible for the depletion of NADPH (Figure 2E).

### 2.3 Hepatocyte‐Specific Deletion of *Me1* Is Associated with Exacerbated Liver I/R Injury

**Figure 3:**
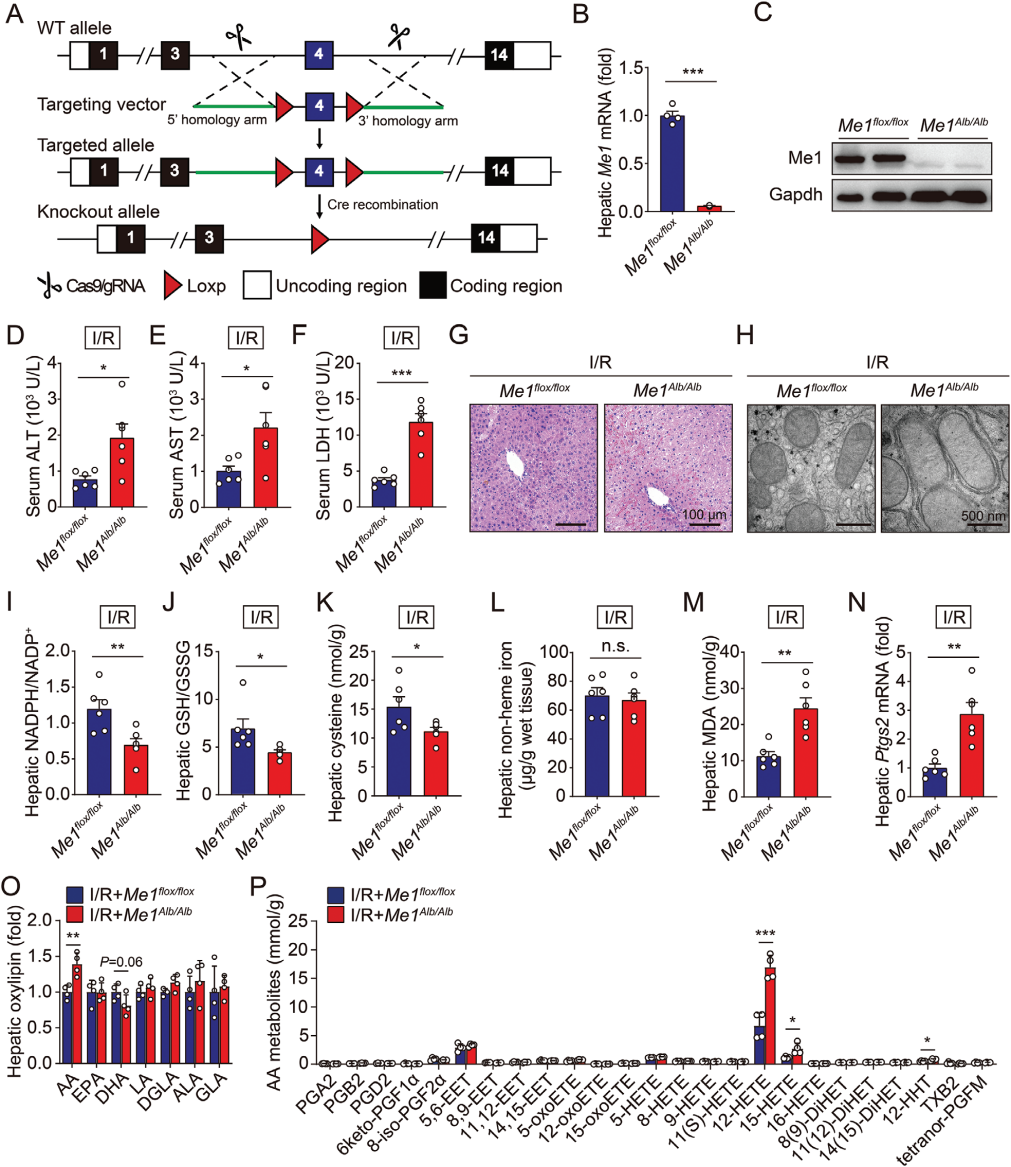
Loss of hepatic Me1 facilitates I/R‐induced ferroptosis and liver damage. A) Schematic diagram depicting the strategy used to generate conditional Me1 knockout mice. B) Hepatic levels of *Me1* mRNA were measured in Me1^flox/flox^ and Me1^Alb/Alb^ mice. *n* = 6 per group. C) Western blot analysis of hepatic Me1 protein in Me1^flox/flox^ and Me1^Alb/Alb^ mice. D–F) Serum levels of ALT (D), AST (E), and LDH (F) were measured in Me1^flox/flox^ and Me1^Alb/Alb^ mice subjected to I/R injury. *n* = 6 per group. G) Representative H&E‐stained liver sections from Me1^flox/flox^ and Me1^Alb/Alb^ mice subjected to I/R injury. H) Electron micrographs showing mitochondria in liver tissue obtained from Me1^flox/flox^ and Me1^Alb/Alb^ mice subjected to I/R injury. I–K) Hepatic levels of NADPH (I), GSH (J), and cysteine (K) were measured in Me1^flox/flox^ and Me1^Alb/Alb^ mice subjected to I/R injury. *n* = 6 per group. L,M) Hepatic contents of non‐heme iron (L) and MDA (M) were measured in Me1^flox/flox^ and Me1^Alb/Alb^ mice subjected to I/R injury. *n* = 6 per group. N) Hepatic levels of *Ptgs2* mRNA were measured in Me1^flox/flox^ and Me1^Alb/Alb^ mice subjected to I/R injury. *n* = 6 per group. O,P) Summary of hepatic oxylipins (O) and arachidonic acid (AA) metabolites (P) were measured in Me1^flox/flox^ and Me1^Alb/Alb^ mice subjected to I/R injury. *n* = 4 per group. Significance was calculated by Student's *t*‐test; *p < 0.05, **p < 0.01, ***p < 0.001.

In light of the significant correlation between suppressed Me1 expression and levels of liver injury, we generated a conditional knockout mouse model to evaluate the specific roles of ME1 in hepatic I/R injury. Exon 4 of *Me1* gene was deleted via the Cre‐LoxP system using a Cre recombinase driven by the serum albumin (Alb) gene promoter (**Figure** **3**A). Hepatocyte‐specific *Me1* deletion (*Me1^Alb/Alb^*) mice were born at the expected Mendelian ratio and were viable. Quantitative real‐time PCR analysis suggested that *Me1* expression was reduced by ≈90% in the liver (Figure 3B), and this was also validated by western blotting result (Figure 3C). When we examined the phenotype of *Me1^Alb/Alb^* mice at 2 months of age, there were no pathological changes in the liver, indicating that deleting *Me1* in hepatocyte is not sufficient to induce liver injury in vivo (Figure S2, Supporting Information).

We therefore investigated the role of ME1 in I/R‐induced hepatic ferroptosis. Compared to *Me1^flox/flox^* littermates, *Me1^Alb/Alb^* mice experienced more severe liver damage following I/R treatment, as measured by serum levels of the enzymes alanine aminotransferase (ALT), aspartate aminotransferase (AST), and lactate dehydrogenase (LDH) (Figure 3D–F). Consistent with these results, histological observation confirmed the increased susceptibility to hepatic I/R injury in *Me1^Alb/Alb^* mice (Figure 3G). With respect to ultrastructural architecture, electron microscopy revealed that I/R‐treated *Me1^Alb/Alb^* mice have swollen mitochondria in hepatocytes, with reduced cristae density (Figure 3H).

Meanwhile, *Me1^Alb/Alb^* livers show more serious deficiency of NADPH, GSH, and cysteine (Figure 3I–K and Figure S3, Supporting Information). Although no changes of hepatic iron contents were observed (Figure 3L), we found higher levels of both malondialdehyde (MDA), an end product of lipid peroxidation, and *Ptgs2* expression, an in vivo biomarker for ferroptosis, in livers of I/R‐treated *Me1^Alb/Alb^* mice (Figure 3M,N). In addition, we measured hepatic oxylipins, which are derived from oxidation of polyunsaturated fatty acids, in I/R‐treated control and *Me1^Alb/Alb^* mice. As shown in Figure 3O,P and Figure S4, Supporting Information, we found significantly increased levels of the oxidized arachidonic acid (AA) metabolites, especially 12‐HETE and 15‐HETE. Together, these results offer compelling evidence for an anti‐ferroptotic role of ME1 during hepatic I/R injury.

### 2.4 L‐Malate Supplementation Reduces I/R‐Induced Hepatic Ferroptosis in Mice

**Figure 4:**
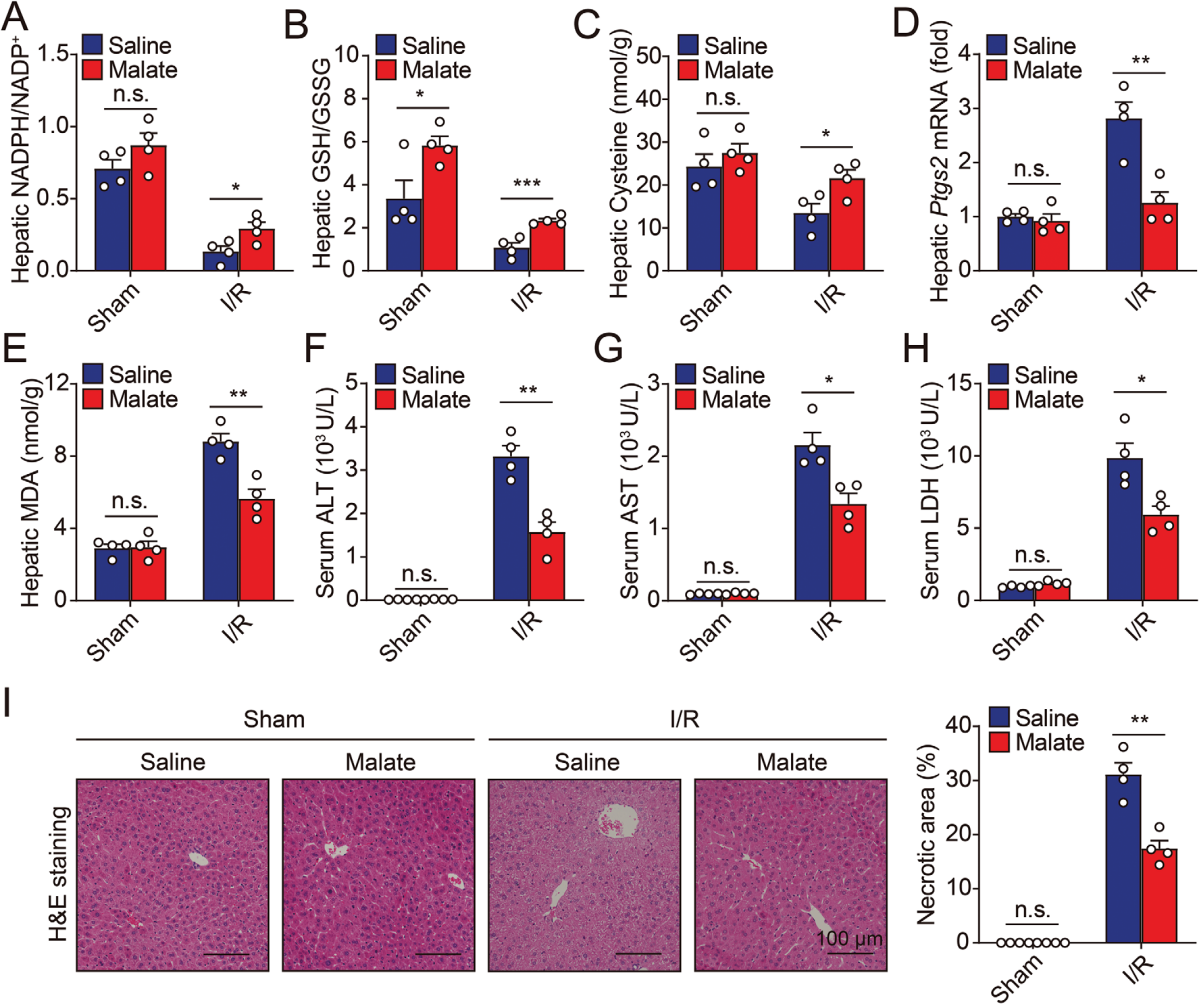
Malate supplementation protects against hepatic ferroptosis during I/R injury. A,B) Ratios of NADPH/NADP^+^ (A) and GSH/GSSG (B) were measured in the livers of sham‐ or I/R‐treated mice with or without malate supplementation. *n* = 4 per group. C) Hepatic cysteine contents were measured in sham‐ or I/R‐treated mice with or without malate supplementation. *n* = 4 per group. D,E) Hepatic *Ptgs2* mRNA (D) and MDA levels (E) were measured in sham‐ or I/R‐treated mice with or without malate supplementation. *n* = 4 per group. F–H) Serum levels of ALT (F), AST (G), and LDH (H) were measured in sham‐ or I/R‐treated mice with or without malate supplementation. *n* = 4 per group. I) Representative H&E‐stained liver sections (left) and quantitative data (right) from sham‐ or I/R‐treated mice with or without malate supplementation. *n* = 4 per group. Significance was calculated by Student's *t*‐test; *p < 0.05, **p < 0.01, n.s. = not significant.

Next, we tested whether malate, the substrate of ME1, could protect against I/R‐induced hepatic ferroptosis and injury. As expected, supplementation of mice with L‐malate, the natural and biologically active isomer contained in food, significantly restored hepatic levels of NADPH, GSH, and cysteine in mice subjected to I/R (**Figure** **4**A–C and Figure S5, Supporting Information). Additionally, I/R‐induced hepatic ferroptosis was also suppressed by L‐malate supplementation (Figure 4D,E). Finally, we confirmed that supplementation with L‐malate markedly attenuated liver injury of I/R‐treated mice (Figure 4F–I). On the other hand, malate supplementation could not ameliorate liver injury in I/R‐treated *Me1^Alb/Alb^* mice, supporting that activation of ME1 is the key target involved (Figure S6, Supporting Information). Taken together, these data indicate the therapeutic potential of L‐malate administration for treating hepatic I/R injury in clinical practice.

### 2.5 Pten Regulates *Me1* Expression during I/R Injury via Akt/mTOR Pathway

**Figure 5:**
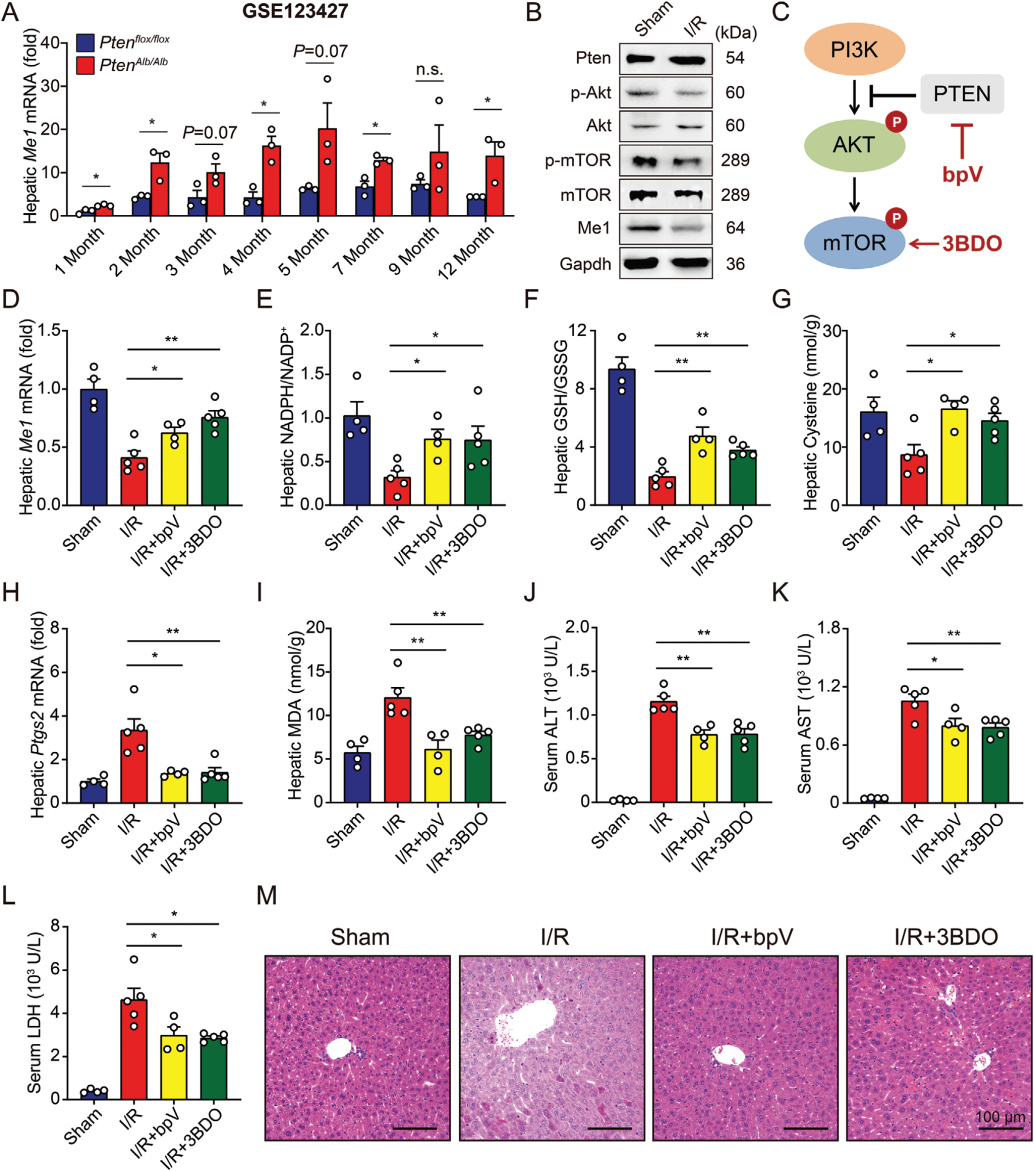
Pten/Akt/mTOR pathway regulates hepatic *Me1* expression. A) Hepatic *Me1* expression was measured in Pten^flox/flox^ and Pten^Alb/Alb^ mice subjected to I/R injury at different months old. *n* = 3 per group. B) Immunoblots of hepatic Pten, p‐Akt, Akt, p‐mTOR, mTOR, and Me1 were analyzed in mice with sham or I/R injury. C) Schematic diagram depicting the targets of bpV and 3BDO. D) Hepatic levels of *Me1* mRNA were measured in I/R‐treated mice with bpV or 3BDO injection. *n* = 4, 5, 4, 5. E,F) Ratios of NADPH/NADP^+^ (E) and GSH/GSSG (F) were measured in the livers of I/R‐treated mice with bpV or 3BDO injection. *n* = 4, 5, 4, 5. G) Hepatic cysteine contents were measured in I/R‐treated mice with bpV or 3BDO injection. *n* = 4, 5, 4, 5. H,I) Hepatic *Ptgs2* mRNA (H) and MDA levels (I) were measured in I/R‐treated mice with bpV or 3BDO injection. *n* = 4, 5, 4, 5. J–L) Serum levels of ALT (J), AST (K), and LDH (L) were measured in I/R‐treated mice with bpV or 3BDO injection. *n* = 4, 5, 4, 5. M) Representative H&E‐stained liver sections from indicated mice. Significance was calculated by one‐way ANOVA; *p < 0.05, **p < 0.01, n.s. = not significant.

Previous studies have highlighted the importance of phosphatase and tensin homologue (PTEN)‐mediated pathway in hepatic I/R injury.^[^ ^15^, ^16^ ^]^ We mined an open dataset (Gene Expression Omnibus dataset GSE123427) and compared the expression levels of *Me1* mRNA between wild‐type and hepatocyte‐specific Pten deletion (*Pten^Alb/Alb^*) mice. As shown in **Figure** **5**A, the hepatic expression of *Me1* was significantly increased in the absence of Pten at most age groups, indicating a mechanism by which PTEN regulates ME1 expression in the liver. Interestingly, we tested ferroptosis‐related genes in the same dataset and the results showed the expression of *Gpx4* was significantly increased in the *Pten*‐deficient livers, which strongly suggested that the impact of PTEN on ferroptosis is through ME1‐mediated NAPDH production (Figure S7, Supporting Information).

Generally, the function of PTEN is realized through its inhibition of the PI3K/Akt/mTOR signaling pathway which controls cell growth and metabolism.^[^ ^17^ ^]^ Then, we found that hepatic I/R treatment reduced western blot‐assisted expression of phosphorylated Akt and mTOR, in line with its effect on hepatic Me1 expression (Figure 5B). To further confirm the significance of PTEN‐mediated signaling in I/R‐induced hepatic ferroptosis, wild‐type mice were pretreated with either a PTEN inhibitor (bpV) or a mTOR activator (3BDO) before receiving liver surgery (Figure 5C and Figure S8, Supporting Information). First, both bpV and 3BDO treatment significantly increased *Me1* expression in the I/R‐treated livers (Figure 5D), and subsequently restored hepatic levels of NADPH, GSH, and cysteine (Figure 5E–G and Figure S9, Supporting Information). Finally, blocking Pten or activating mTOR also prevented the I/R‐induced ferroptosis (Figure 5H,I) and tissue injury (Figure 5J–M) in the murine liver.

We additionally tested the effect of another mTOR activator L‐leucine in the model. As shown in Figure S10, Supporting Information, leucine treatment successfully increased Me1 expression and suppressed I/R‐induced ferroptosis and liver injury. Taken together, we confirmed that mTOR is the upstream molecular mediating Me1 expression and further ferroptosis in liver I/R injury.

### 2.6 Hepatic Over‐Expression of Srebp1 Provides Liver Protection against I/R Injury

**Figure 6:**
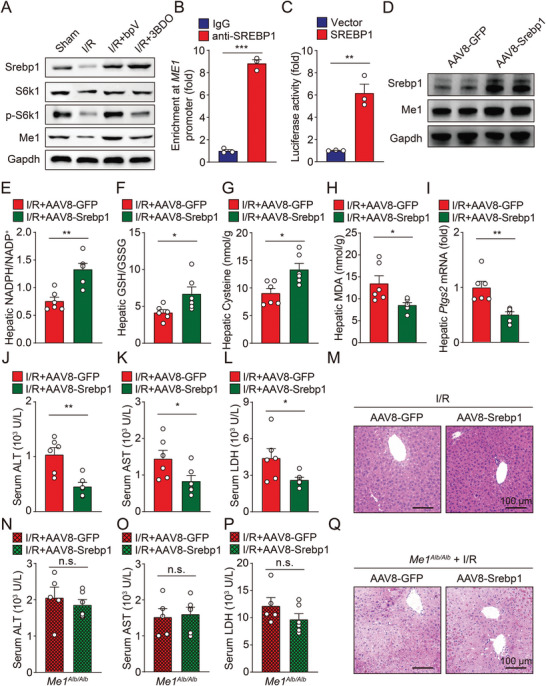
Srebp1 is critical for mTOR signaling to activate *Me1* transcription, maintain NADPH homeostasis, and suppress ferroptosis in liver. A) Immunoblots of hepatic Srebp1, S6K1, p‐S6K1, and Me1 were measured in I/R‐treated mice with bpV or 3BDO injection. B) Enrichment of SREBP1 binding to the ME1 promoter was measured by ChIP‐qPCR analysis in HEK293 cells. C) Luciferase reporter assay in HEK293 cells transfected with a ME1‐luciferase construct in the presence of SREBP1 or empty vector alone. D) Immunoblots of hepatic Srebp1 and Me1 in mice treated with AAV8‐Srebp1 or AAV8‐GFP. E,F) Ratios of NADPH/NADP^+^ (E) and GSH/GSSG (F) were measured in the livers of I/R‐treated mice with or without AAV‐mediated *Srebp1* overexpression. G) Hepatic cysteine contents were measured in I/R‐treated mice with or without AAV‐mediated *Srebp1* overexpression. H,I) Hepatic *Ptgs2* mRNA (H) and MDA levels (I) were measured in I/R‐treated mice with or without AAV‐mediated *Srebp1* overexpression. *n* = 6 per group. J–L) Serum levels of ALT (J), AST (K), and LDH (L) were measured in I/R‐treated mice with or without AAV‐mediated *Srebp1* overexpression. *n* = 6 per group. M) Representative H&E‐stained liver sections from indicated mice. N–P) Serum levels of ALT (N), AST (O), and LDH (P) were measured in I/R‐treated *Me1^Alb/Alb^* mice with or without AAV‐mediated *Srebp1* overexpression. *n* = 5, 6. Q) Representative H&E‐stained liver sections from indicated mice. Significance was calculated by Student's *t*‐test; *p < 0.05, **p < 0.01, ***p < 0.001.

Sterol regulatory element (SRE)‐binding protein 1 (SREBP1) is a master regulator of lipogenesis and a downstream target of Akt/mTOR signaling pathway.^[^ ^18^, ^19^ ^]^ S6 kinase 1 (S6K1), a downstream effector of mTOR, was reported as the candidate kinase for specific proteolytic processing of SREBP1 precursor in hepatocytes.^[^ ^20^ ^]^ Interestingly, SREBP1 transgenic mice have marked elevation in hepatic *Me1* expression.^[^ ^21^ ^]^ In addition, hepatic expression of Srebp1 as well as phosphorylated S6k1 were both significantly reduced after I/R treatment, which could be prevented by PTEN inhibition or mTOR activation (**Figure** **6**A).

Although no authentic SRE site was found in ME1 promoter, there are several SRE half sites that may involve in the interaction with SREBP1. Indeed, chromatin immunoprecipitation (ChIP)‐qPCR analysis found that SREBP1 could directly bind to the *ME1* promoter (Figure 6B). Consistent with previous report,^[^ ^22^ ^]^ overexpression of SREBP1 strongly activated the luciferase‐driven *ME1* promoter in vitro, further supported that SREBP1 is the key transcription factor regulated by mTOR and directly controlling *ME1* transcription (Figure 6C).

Then, we generated a recombinant adeno‐associated virus serotype‐8 (AAV8) vector carrying murine *Srebp1* gene and infused it into wild‐type mice. Compared to AAV8‐green fluorescent protein (GFP) control mice, mice transduced with AAV8‐Srebp1 had significantly elevated Me1 expression in the liver (Figure 6D and Figure S11, Supporting Information). After hepatic I/R operation, overexpressing Srebp1 in hepatocytes successfully restored hepatic NADPH/NADP^+^ ratio, GSH/GSSG ratio, and cysteine levels (Figure 6E–G and Figure S12, Supporting Information) and suppressed ferroptosis (Figure 6H,I). Finally, hepatic overexpression of Srebp1 rendered wild‐type livers more resistant to I/R‐induced injury (Figure 6J–M), but it had no protective effect in *Me1*‐deficient mice (Figure 6N–Q), further conforming that SREBP1 suppresses hepatic ferroptosis in a ME1‐dependent manner.

## 3 Discussion

**Figure 7:**
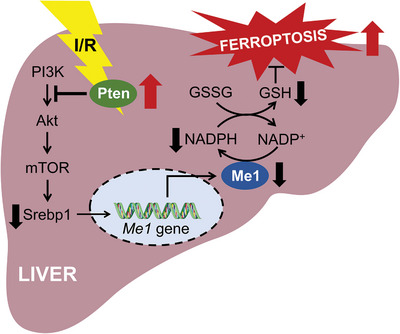
Working model depicting how ME1 is regulated and suppresses ferroptosis in hepatic I/R injury. The maintaining of *ME1* gene expression in the liver is governed by the canonical PI3K/AKT/mTOR pathway. mTOR activates the transcription factor SREBP1, which could bind to the promoter region of *ME1* gene and lead to transactivation. ME1 replenishes the intracellular reducing equivalent NADPH and contributes to maintain redox homeostasis. Under I/R procedure, PTEN activation suppresses the PI3K/AKT/mTOR pathway, resulting in downregulation of *ME1*. As consequence, depleted NADPH levels significantly increase the susceptibility to hepatic ferroptosis, leading to aggravated I/R injury.

Hepatic I/R injury is responsible for morbidity and mortality in patients undergoing liver surgery. Ferroptosis is considered to be a key event in the pathophysiological process. The current study is designed to pinpoint the molecular mechanisms by which I/R facilitates hepatic ferroptosis and ensuing liver injury. To make progress on this front, we focused on marked reduction in NADPH during hepatic I/R injury and then performed a targeted gene screen in the liver of murine model. This approach revealed a prominent decrease in hepatic Me1 expression. We found a profound protective role for ME1 against hepatic ferroptosis, whereby mice with hepatocyte‐specific deletion of Me1 experienced higher susceptibility to I/R‐induced liver injury. In support of these findings, supplementation of L‐malate restored NADPH and alleviated hepatic ferroptosis. Mechanistically, we found that ME1 is a SREBP1 target gene in the liver and regulated upstream by PTEN/Akt/mTOR pathway (**Figure** **7**).

Ferroptosis is governed by the cyst(e)ine/GSH/GPX4 antioxidant axis, which is fueled by NADPH.^[^ ^23^ ^]^ The decrease of NADPH may be a biomarker predicting for ferroptosis sensitivity,^[^ ^10^ ^]^ and depletion of NADPH directly sensitizes cancer cells to ferroptosis, further supporting NADPH as an important propellant for the ferroptosis defense system.^[^ ^24^ ^]^ However, NADPH may be a double‐edged sword in ferroptosis regulation, since it is also a substrate for NOXs. NOXs are transmembrane enzymes that promote ferroptosis by catalyzing the one‐electron reduction of molecular oxygen to superoxide anion.^[^ ^25^, ^26^ ^]^ It is still in doubt that whether the anti‐ferroptotic role of NADPH overweighs its potential pro‐ferroptotic function; but in the present study, the involvement of NOXs was excluded by transcriptional analysis of gene expression.

The PPP is an important glucose metabolism pathway, in which NADPH is produced by both glucose‐6‐phosphate dehydrogenase (G6PD) and 6‐phosphogluconate dehydrogenase.^[^ ^27^ ^]^ Knockdown of G6PD has been proven to inhibit ferroptosis in hepatocellular carcinoma cell lines.^[^ ^28^ ^]^ In addition to the oxidative PPP, quantitative flux analysis has revealed that intracellular NADP^+^ could also be recycled to NADPH by folate‐mediated one‐carbon metabolism, malic enzymes, and isocitrate dehydrogenases.^[^ ^29^ ^]^

Malic enzymes are a class of oxidative decarboxylases required for the reversible conversion of malate to pyruvate, a key connector linking glycolysis to the tricarboxylic acid cycle. In mammalian cells, the family consists of three isoforms: a cytosolic NADP^+^‐dependent isoform (ME1), a mitochondrial NAD^+^‐dependent isoform (ME2), and a mitochondrial NADP^+^‐dependent isoform (ME3).^[^ ^13^ ^]^ Among them, ME1 is the most abundant isoform, accounting for approximately two‐thirds of malic enzyme expression.^[^ ^30^ ^]^ ME1 is frequently overexpressed in cancer cells, and knockdown of ME1 expression could induce ROS accumulation and suppress cell viability in either gastric or breast cancer.^[^ ^31^, ^32^ ^]^

A recent study by Brashears et al. showed that synovial sarcoma lacks ME1 expression which drives decrease in intracellular NADPH and GSH levels.^[^ ^33^, ^34^ ^]^ Interestingly, ME1 deficiency render synovial sarcoma cells sensitive to erastin‐induced ferroptosis, but resistant to GSH depletion‐induced cell death. Reprogramming of redox homeostasis in the context of ME1 absence may explain how these cells adapt to be less dependent on GSH. But whether similar mechanism works in our mouse model remains to be further investigated. Notably, ME1‐deficient synovial sarcoma cells were also found to have intercellular iron accumulation. However, our data clearly showed that hepatic iron levels were maintained in I/R‐treated *Me1^Alb/Alb^* mice. It is possible that ME1 function in the regulation of iron metabolism is context dependent as well.

To the best of our knowledge, there is no reliable activator for ME1, thus we chose to supplement mice with its substrate L‐malate. According to previous studies, L‐malate could strengthen antioxidative activity and improve liver function of aged animals.^[^ ^35^ ^]^ Tang et al. reported that pretreatment with L‐malate significantly ameliorated myocardial I/R injury with suppressed inflammatory response.^[^ ^36^ ^]^ Non‐apoptotic forms of regulated cell death can release damage‐associated molecular patterns and trigger sterile inflammation, referred to as necroinflammation.^[^ ^37^ ^]^ Therefore, it is reasonable to speculate that L‐malate inhibited ferroptosis in the model of cardiac injury.

Importantly, these benefits of L‐malate are highly clinically relevant. Since its discovery a decade ago, the ability to direct suppression toward ferroptosis is very attractive for clinical management of tissue injuries. Although several small molecules have been proposed targeting ferroptosis in cell lines or animal models, to translate them to clinical trials is difficult.^[^ ^38^ ^]^ In contrast, L‐malate was registered as a safe, nontoxic, harmless, and edible organic acid by the US Food and Drug Administration in 1967, suggesting its potential in clinical practice. Our findings provide a preclinical basis for purposing of L‐malate into clinical trials for hepatic I/R injury management.

Relevant to regulatory mechanism, this study reveals that activation of mTOR by agonists or inhibition of PTEN restores hepatic ME1 expression and suppresses ferroptosis via downstream target SREBP1. PTEN mainly acts as a phosphatase which dephosphorylates phosphatidylinositol triphosphate and thus negatively regulates PI3K‐dependent Akt/mTOR signaling.^[^ ^17^ ^]^ It was first discovered as a tumor suppressor, and hepatocyte‐specific *Pten* deficiency resulted in hepatocellular carcinomas in mice.^[^ ^39^, ^40^ ^]^ However, emerging evidence has highlighted the role of PTEN in protecting liver against acute injury.^[^ ^41^, ^42^ ^]^ With the help of transcriptome sequencing, we hypothesized that PTEN pathway negatively regulates hepatic *ME1* transcript and thus contributes to I/R‐induced ferroptosis. Our results regarding the PTEN inhibitor are in line with this important hypothesis and back up previously published data.^[^ ^15^, ^16^ ^]^

Notably, a series of recent studies indicated intriguing interplays between mTOR signaling and ferroptosis.^[^ ^43^ ^]^ mTOR exists in two distinct multiprotein complexes, namely mTOR complex 1 (mTORC1) and mTORC2, and only mTORC1 is sufficient to stimulate SREBP1 activity, which depends on the phosphorylation of S6K1.^[^ ^44^ ^]^ Phosphorylated S6K1 initiates the proteolytic activation of SREBP1 precursor located on endoplasmic reticulum and increases translocation of the processed protein into the nucleus.^[^ ^20^, ^45^ ^]^ Yi et al. showed that mTORC1/SREBP1 axis could protect cultured cancer cells from ferroptosis through stearoyl‐CoA desaturase‐1, an enzyme converting saturated fatty acids to monounsaturated fatty acids.^[^ ^46^ ^]^ However, another recent study showed that mTOR overactivation may promote ferroptosis via protein synthesis and subsequently depletes intracellular amino acid pools.^[^ ^47^ ^]^ Thus, mTOR‐mediated regulation of ferroptosis is likely to be context‐dependent, which is consistent with its pleiotropic role o in cellular metabolism.^[^ ^43^ ^]^

Nevertheless, we could not completely exclude the potential of other mechanisms being involved in regulation of ME1‐mediated ferroptosis in the liver. For example, AMPK is classic upstream factor for mTOR and could inhibit ferroptosis.^[^ ^48^ ^]^ Importantly, AMPK activation could reduce the damage of I/R to the liver.^[^ ^49^ ^]^ But previous studies pointed out that the AMPK‐mediated pathway may function primarily in macrophages instead of hepatocytes.^[^ ^50^, ^51^, ^52^ ^]^ A better understanding of the regulatory network at single‐cell level is key for further investigation.

In summary, our findings indicate that suppressed ME1 expression in hepatocytes is associated with hepatic vulnerability to I/R injury through a pro‐ferroptotic mechanism dependent on NADPH production. From a mechanistic perspective, we also provide compelling evidence that PTEN/mTOR/SREBP1 signaling regulates hepatic ME1 expression in ferroptosis induction. Hence, further identification of specific ME1 activators as inhibitors of ferroptosis could have direct translational implications for clinical therapy of liver injury.

## 4 Experimental Section

### Experimental Animals

All experiments involving animals were performed in accordance with the National Institutes of Health's Guide for the Care and Use of Laboratory Animals and were approved by the Animal Care and Use Committee of Hangzhou Normal University (No. HSD20211103). Wild‐type C57BL/6J mice aged 8 weeks were purchased from GemPharmatech. The *Me1*‐floxed (*Me1^flox/flox^*) mice and *Alb‐Cre* transgenic mice were produced by Shanghai Model Organisms Center.

### Hepatic I/R Injury Model

Mice were placed in a supine position on a heating pad (37 °C). Midline laparotomy was performed to expose the liver under isoflurane anesthesia. Ischemia was induced in the left liver lobes by clamping the branch of the portal triad using a microaneurysm clamp. This procedure results in a segmental ischemia which manifests as a decolorization of the ischemic lobes. During ischemia, the abdomen was covered with sterile soaked tissues to minimize evaporative loss. After 1 h, the clamp was removed to initiate reperfusion for 6 h. Sham‐operated animals underwent the same procedures without liver ischemia.

### In Vivo Drug Administration

L‐Malate (M7397, Sigma‐Aldrich, 10 mg kg^−1^), L‐Leucine (L8912, Sigma‐Aldrich, 100 mg kg^−1^), bpV (S8651, Selleck, 1 mg/kg body weight), or 3BDO (S8317, Selleck, 10 mg/kg body weight) were administered intraperitoneally 30 min before ischemia surgery and injected intravenously 5 min prior to reperfusion, respectively.

### Overexpression of Hepatic *Srebp1*

AAV8 under the control of the alpha‐1 antitrypsin promoter was used to specifically overexpress *Srebp1* (AAV8­*Srebp1*) in hepatocytes of mouse livers. AAV8‐GFP was used as the control. Approximately 1 × 10^11^ genome copies of AAV vectors per mouse were injected via tail vein. 3 weeks after injection, the mice were anesthetized for hepatic I/R surgery.

### Measurement of Liver Damage

Serum enzymes, including ALT, AST, and LDH, were measured using an automatic biochemical analyzer (Sysmex). The levels of serum enzymes were assayed according to the instructions provided with the corresponding kits.

### Quantification of Targeted Metabolites

Hepatic levels of GSH, GSSG, NADPH, NADP^+^, and cysteine were measured by ultra‐high performance liquid chromatography‐tandem mass spectrometry (UHPLC‐MS/MS). The platform utilized in the project was an Agilent 1290 Infinity II UHPLC coupled to an Agilent 6470A Triple Quadrupole MS. Oxylipins were analyzed as previously described.^[^ ^53^ ^]^

### Measurement of MDA

Hepatic MDA levels were measured using thiobarbituric acid method by a commercial kit (Solarbio) according to the manufacturer's instructions.

### Measurement of Tissue Iron

Tissue non‐heme iron contents were measured as previously described.^[^ ^54^ ^]^

### Histology

Livers were removed, fixed overnight in 4% paraformaldehyde (pH 7.4), embedded in paraffin, and serially sectioned at 5‐µm thickness. The sections were then stained with hematoxylin and eosin (H&E) for routine histological examination using a light microscope. Representative images were selected based on the value closest to the mean value of each group.

### Immunohistochemistry

The anti‐Me1 antibody used for immunofluorescence staining was purchased from Proteintech (16619‐1‐AP). Images were taken with Olympus fluorescence microscope. ImageJ software (NIH) was used for processing and quantifying the positive areas from those images.

### Transmission Electron Microscopy

The samples were prepared as described previously,^[^ ^53^ ^]^ and were viewed using a H‐7650 (120 kv) transmission electron microscope (Hitachi).

### Measurement of Me1 Activity

Hepatic Me1 activity was measured using an assay kit (BC1125, Solarbio) in accordance with the manufacturer's instructions.

### RNA Isolation and Quantitative Real‐Time PCR

Total RNA was isolated from tissues or cells using Trizol (Pufei), and RNA concentration and purity were measured using a spectrophotometer. RNA was reverse‐transcribed using the PrimeScript RT reagent Kit (Takara) in accordance with the manufacturer's instructions, and quantitative PCR was performed using a CFX96 Real‐Time System (Bio‐Rad) with SYBR Green Supermix (Bio‐Rad) in accordance with the manufacturer's instructions. All reactions were performed in triplicate, and specificity was monitored using melting curve analysis. See Table S1, Supporting Information, for the primers used.

### Western Blotting

Total proteins were extracted from the tissues by homogenization in RIPA buffer containing protease inhibitors. The homogenate was cleared by centrifugation at 4 °C for 30 min at 12 000 rpm, and the supernatant (containing the protein fraction) was collected. Protein concentration in the supernatant was measured using the BCA Protein Assay Kit (Beyotime). A total of 20 mg protein per sample was resolved in a 10–12% SDS‐PAGE gel and transferred to a nitrocellulose membrane. The membranes were blocked with 5% w/v BSA in tris‐buffered saline containing 0.2% Tween‐20, and then incubated at 4 °C overnight with primary antibody. The membranes were then washed and probed with the appropriate horseradish peroxidase‒conjugated secondary antibody and detected using the Pierce ECL System (Thermo Scientific). See Table S2, Supporting Information, for the antibodies used.

### ChIP Assay

ChIP was performed using the Simple Ch‐IP Plus Enzymatic Chromatin IP Kit (Cell Signaling) in accordance with the manufacturer's instructions. Chromatin was prepared from HEK293 cells. Immune complexes containing SREBP1 were enriched using magnetic beads and antibody against SREBP1 (Santa Cruz). IgG (Proteintech) immunoprecipitation was used as a negative control.

### Luciferase Reporter Assay

The following plasmids were purchased from OBiO: pSLenti‐SPEBP1‐3xFLAG and pGL4.10‐ME1 promoter. HEK293 cells were also transfected with an empty vector as a negative control. Luciferase activity was examined by the Dual‐Luciferase Reporter Assay System (Promega) in accordance with the manufacturer's instructions.

### Statistics

Data were analyzed and plots were generated using GraphPad Prism version 8.0, and all summary data were presented as the mean ± SEM. Groups were compared using the two‐tailed Student's *t*‐test (for comparing two groups) or one‐way ANOVA with Tukey's post hoc test (for multi‐group comparisons). Representative images were selected based on the value closest to the mean value of each group. Statistical significance was set at *p* < 0.05.

## Conflict of Interest

The authors declare no conflict of interest.

## Author Contributions

X.F. and F.W. designed the study. X.F., J.Z., Y.L., and Y.S. performed the experiments. J.Y. and F.L. analyzed the data. X.F. drafted the manuscript. J.M., Y.Y., and Z.C. provided critical feedback on the manuscript. All authors read and approved the final manuscript.

## Supporting information

Supporting Information

## Data Availability

The data that support the findings of this study are available from the corresponding author upon reasonable request.
