# Supplementary material for: Malic Enzyme 1 as a Novel Anti‐Ferroptotic Regulator in Hepatic Ischemia/Reperfusion Injury
Source: Adv Sci (Weinh). 2023 Feb 25;10(13):2205436. doi: 10.1002/advs.202205436 (PMC10161122; doi:10.1002/advs.202205436)
Supplement: Supplementary file 1 — Supporting Information [file ADVS-10-2205436-s001.pdf]

ADVANCED SCIENCE
Open Access

Supporting Information

for *Adv. Sci.*, DOI 10.1002/advs.202205436

Malic Enzyme 1 as a Novel Anti-Ferroptotic Regulator in Hepatic Ischemia/Reperfusion Injury

Xuexian Fang, Jiawei Zhang, You Li, Yijing Song, Yingying Yu, Zhaoxian Cai, Fuzhi Lian, Jun Yang, Junxia Min and Fudi Wang**

Supporting Information for

Malic Enzyme 1 as a Novel Anti-ferroptotic Regulator in Hepatic Ischemia/Reperfusion Injury

Xuexian Fang *et al.*

This PDF file includes:

Figures S1-12

Tables S1-2

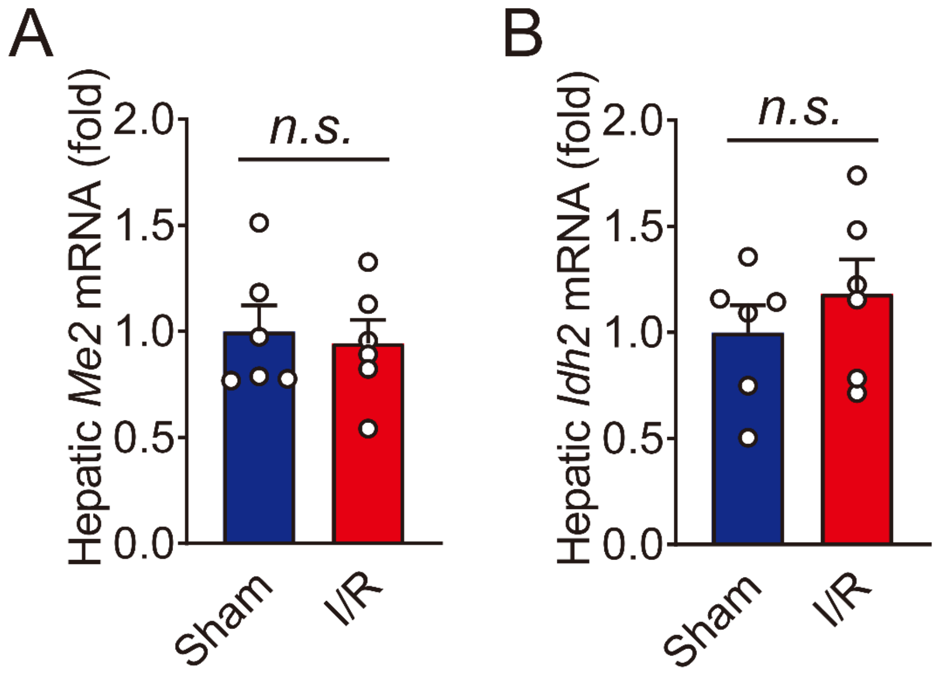

Figure S1. Hepatic *Me2* (A) and *Idh2* (B) mRNA of were measured in mice with sham or I/R injury. Significance was calculated by Student's *t*-test; n.s.=not significant.

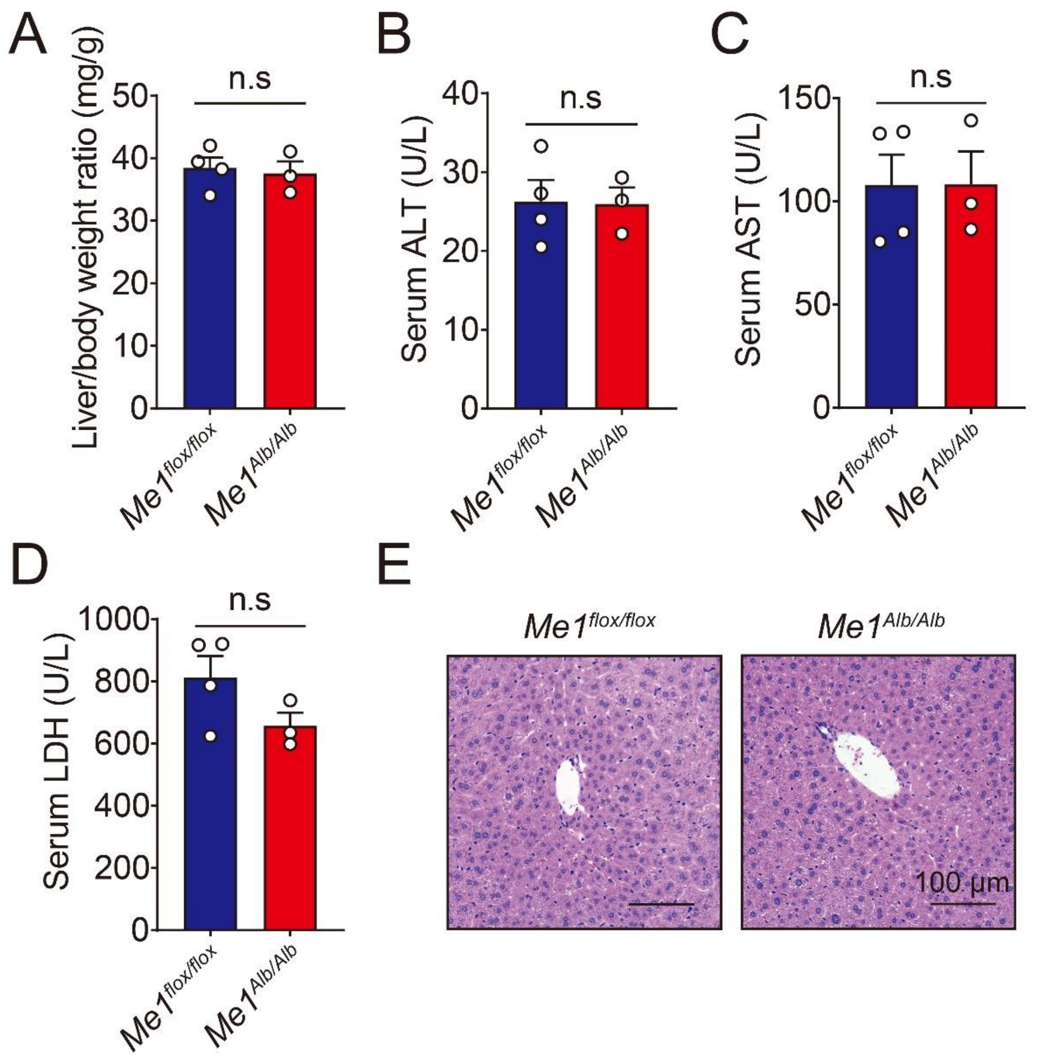

Figure S2. Hepatocyte-specific deletion of *Me1* is not sufficient to induce liver injury in mice. (A-C) Serum levels of ALT (A), AST (B), and LDH (C) were measured in *Me1*$^{flox/flox}$ and *Me1*$^{Alb/Alb}$ mice. (D) Representative H&E-stained liver sections from *Me1*$^{flox/flox}$ and *Me1*$^{Alb/Alb}$ mice. Significance was calculated by Student's *t*-test; n.s.=not significant.

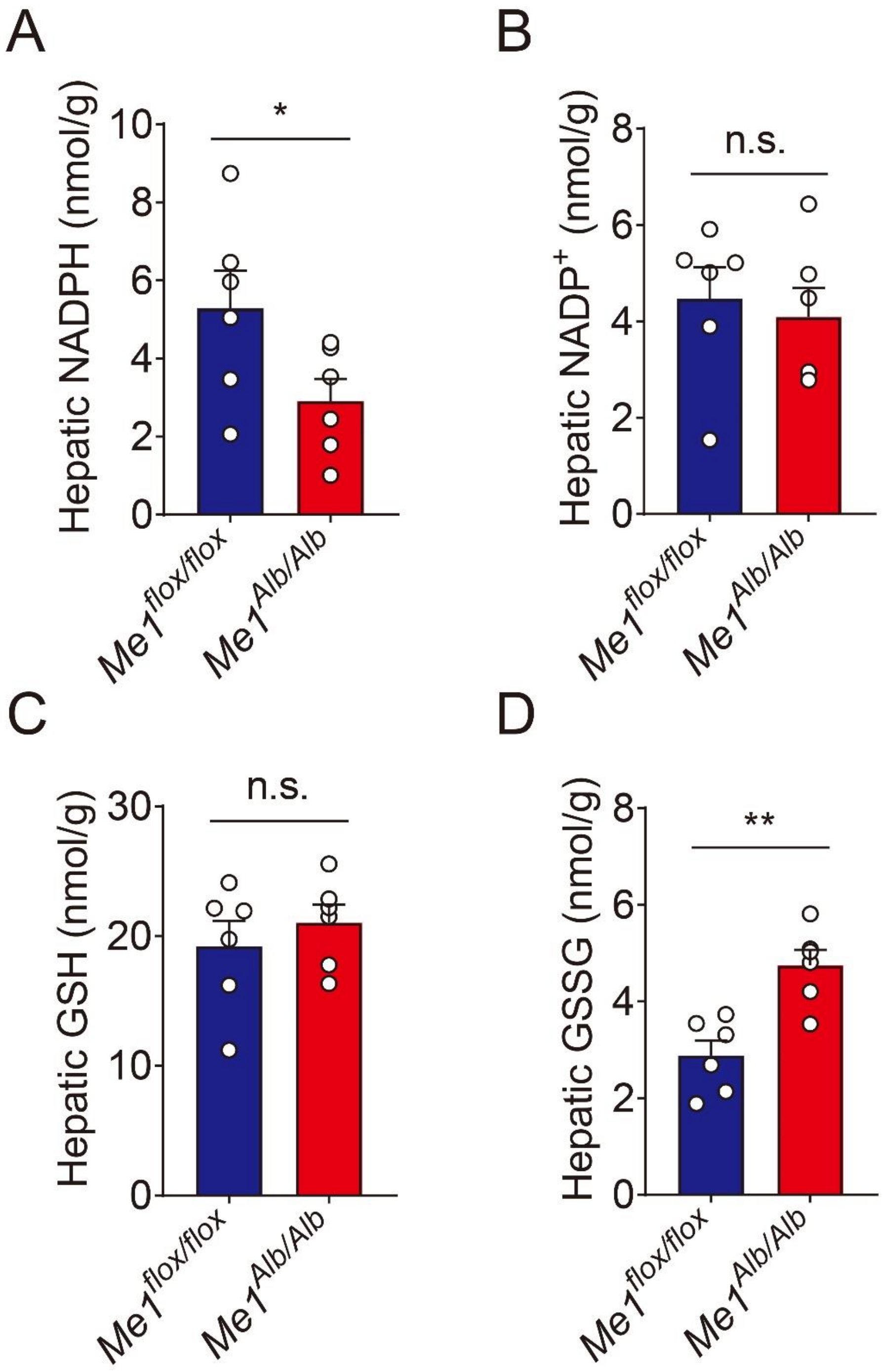

Figure S3. Hepatic levels of NADPH (A), $NADP^+$ (B), GSH (C), and GSSG (D) were measured in *Me1*$^{flox/flox}$ and *Me1*$^{Alb/Alb}$ mice subjected to I/R injury. Significance was calculated by Student's *t*-test; **P*<0.05, ***P*<0.01, ****P*<0.001, n.s.=not significant.

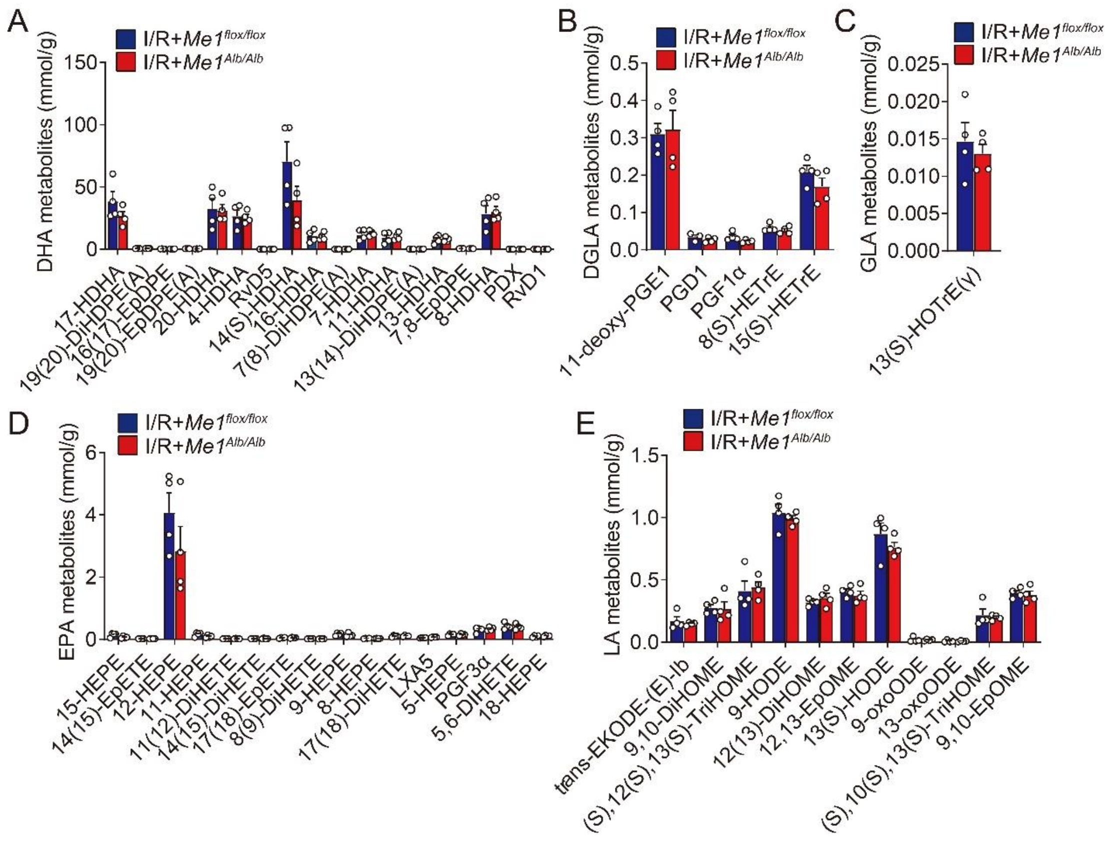

Figure S4. Hepatic docosahexaenoic acid (DHA) metabolites (A), dihomo-γ-linolenic acid (DGLA) metabolites (B), γ-linolenic acid (GLA) metabolites (C), eicosapentaenoic acid (EPA) metabolites (D), linoleic acid (LA) metabolites (E) in *Me1*$^{flox/flox}$ and *Me1*$^{Alb/Alb}$ mice subjected to I/R injury. Significance was calculated by Student’s *t*-test.

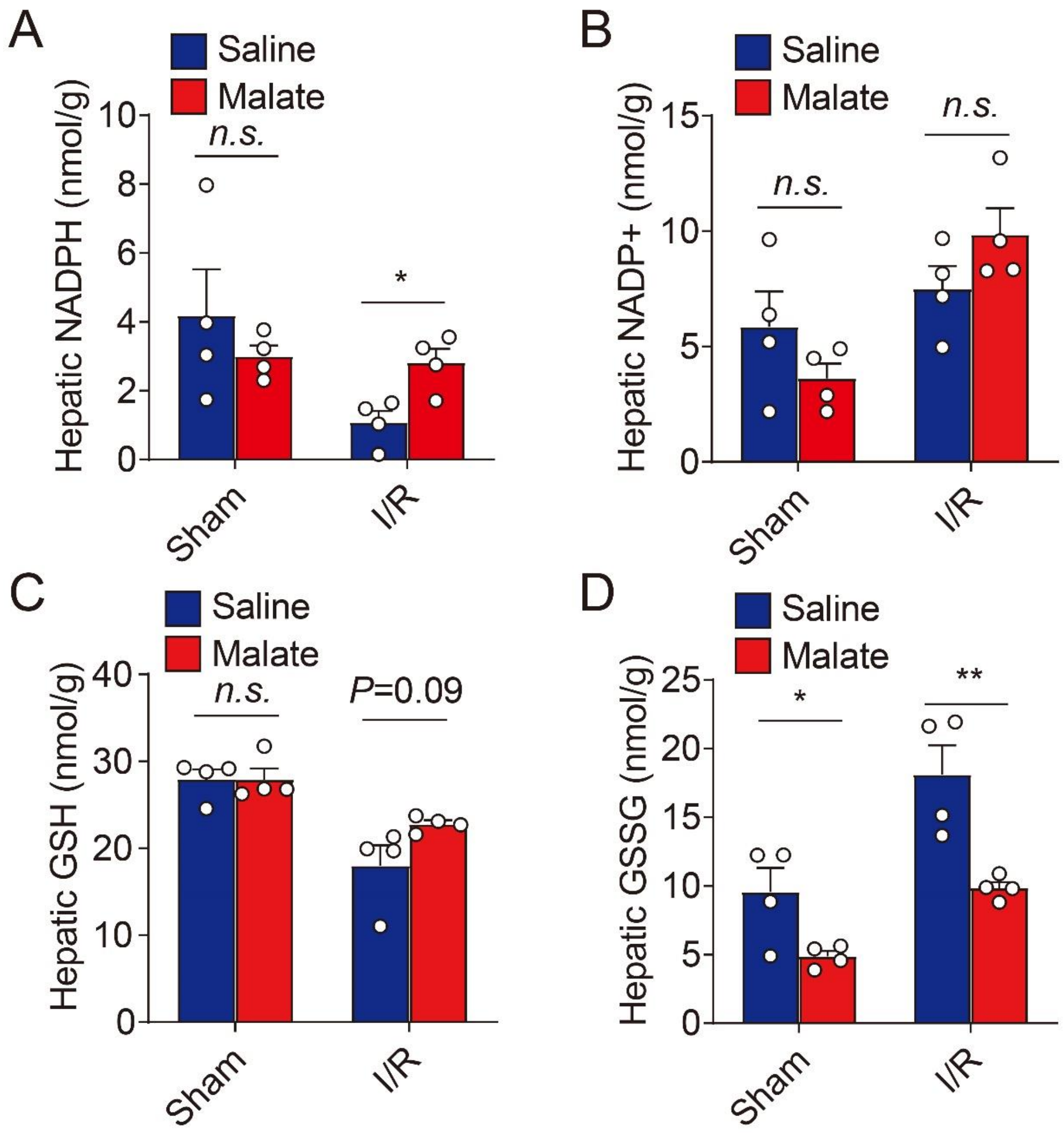

Figure S5. Hepatic levels of NADPH (A), $NADP^+$ (B), GSH (C), and GSSG (D) were measured in sham- or I/R-treated mice with or without malate supplementation. Significance was calculated by Student's *t*-test; *$P<0.05$, **$P<0.01$, ***$P<0.001$, n.s.=not significant.

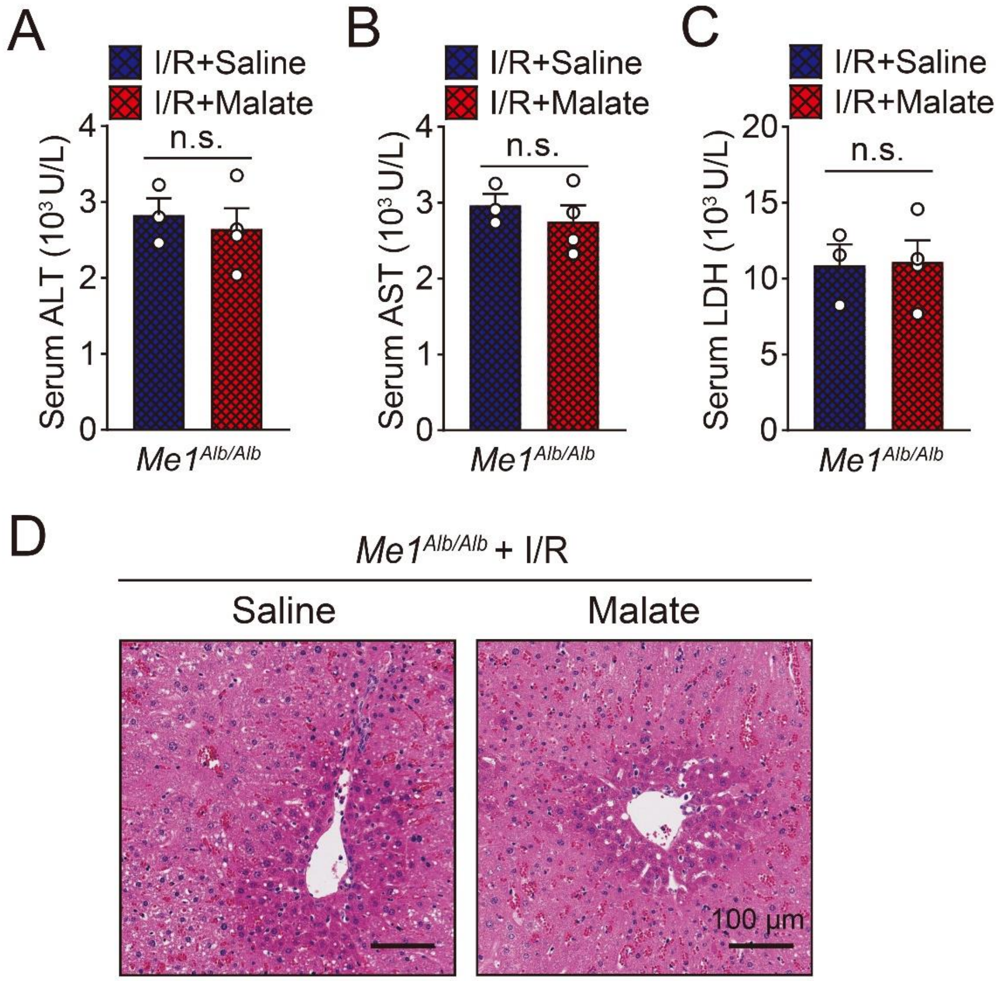

Figure S6. Effect of malate supplementation on *Me1*$^{Alb/Alb}$ mice subjected to I/R injury. **(**A-C) Serum levels of ALT (A), AST (B), and LDH (C) were measured in I/R-treated Me1Alb/Alb mice with or without malate supplementation. (D) Representative H&E-stained liver sections from indicated mice. Significance was calculated by Student's *t*-test; n.s.=not significant.

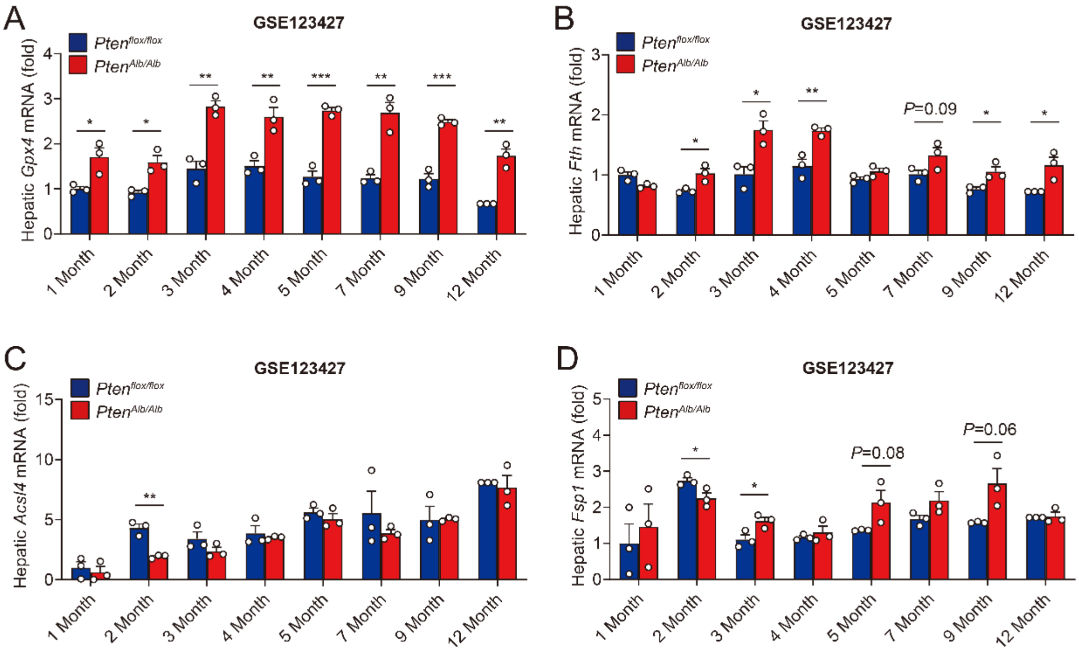

Figure S7. Hepatic expression of *Gpx4* (A), *Fth* (B), *Acsl4* (C), and *Fsp1* (D) were measured in *Pten*$^{flox/flox}$ and *Pten*$^{Alb/Alb}$ mice subjected to I/R injury at different months old. Significance was calculated by Student's *t*-test; **P*<0.05, ***P*<0.01, ****P*<0.001, n.s.=not significant.

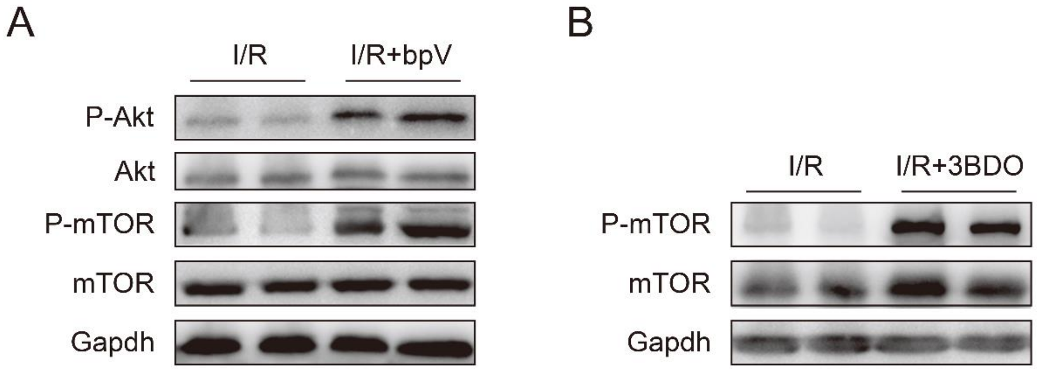

Figure S8. Validation of PTEN inhibitor (bpV) and mTOR activator (3BDO). (A) Immunoblots of hepatic P-Akt, Akt, P-mTOR, and mTOR were measured in I/R-treated mice with or without PTEN inhibitor bpV injection. (B) Immunoblots of hepatic P-mTOR, and mTOR were measured in I/R-treated mice with or without mTOR activator 3BDO injection.

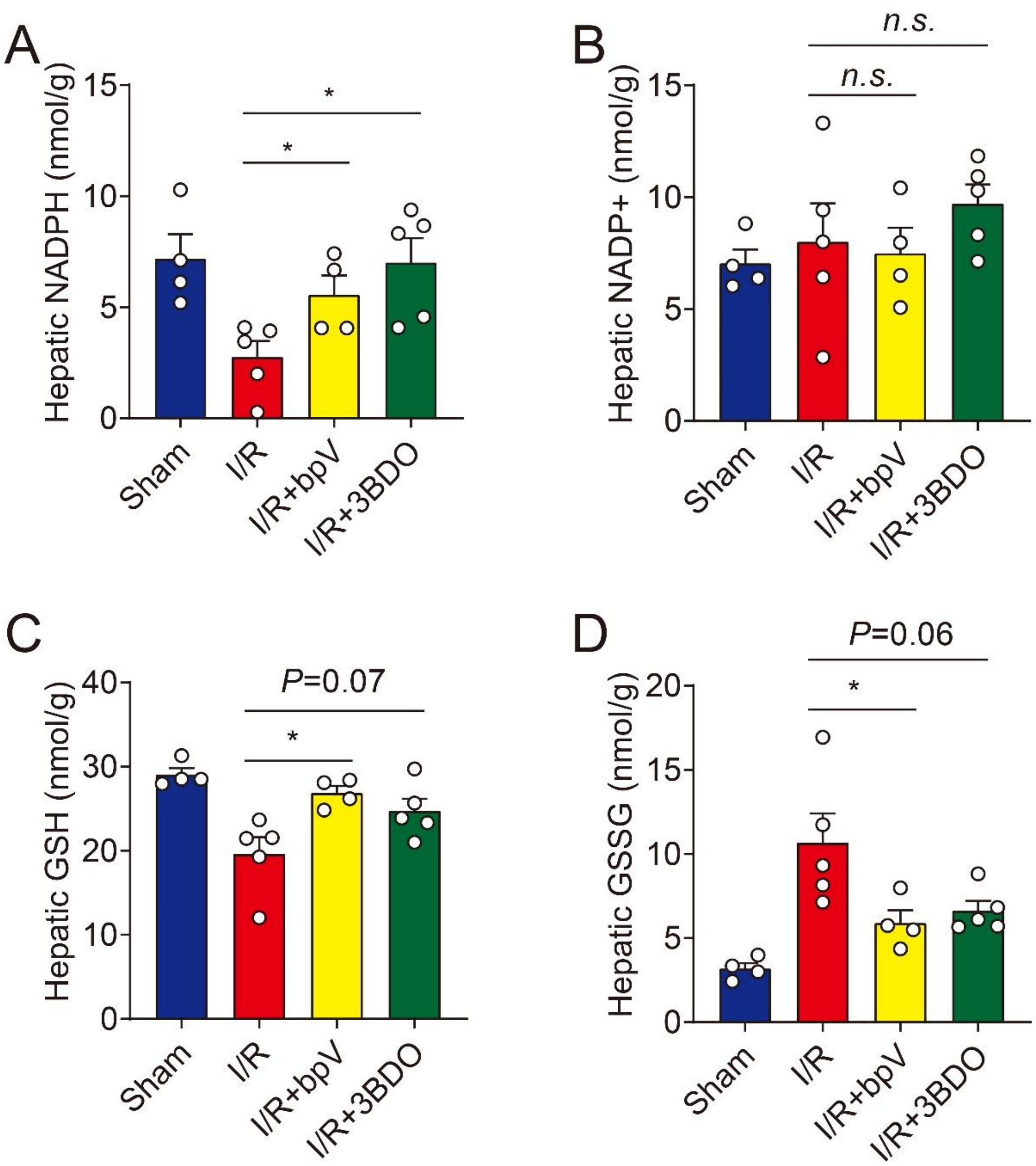

Figure S9. Hepatic levels of NADPH (A), $NADP^+$ (B), GSH (C), and GSSG (D) were measured in I/R-treated mice with bpV or 3BDO injection. Significance was calculated by Student's *t*-test; **P*<0.05, n.s.=not significant.

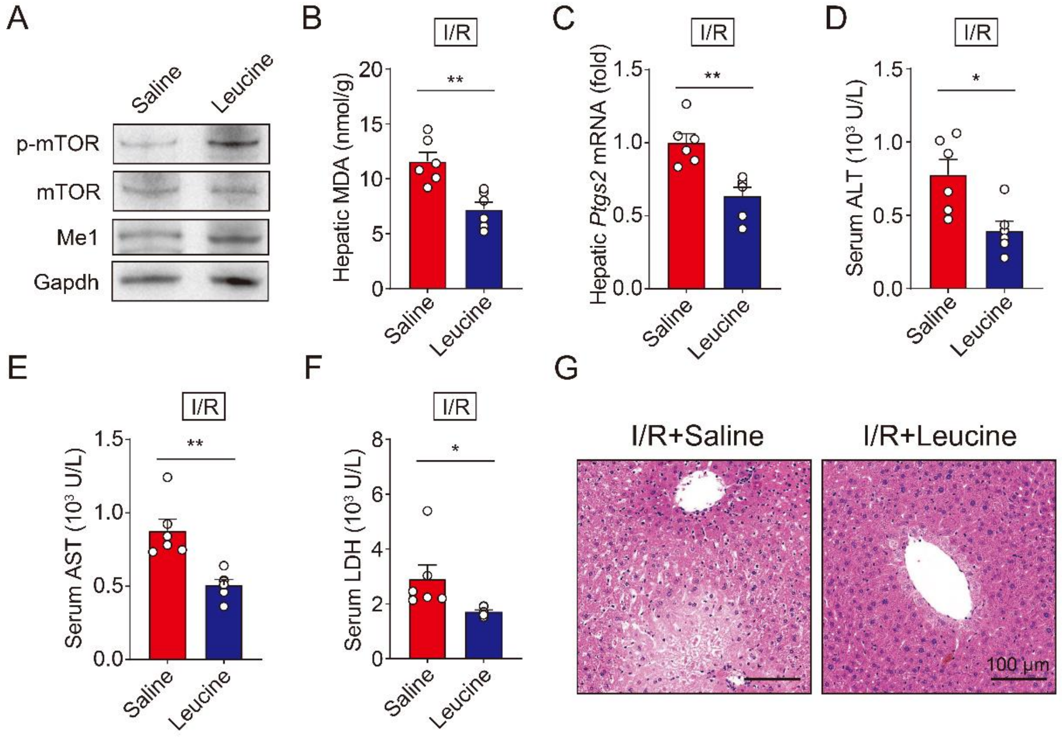

Figure S10. L-leucine supplementation protects against hepatic ferroptosis during I/R injury. (A) Immunoblots of hepatic P-mTOR, mTOR, and Me1 were measured in mice with or without leucine supplementation. (B,C) Hepatic *Ptgs2* mRNA (B) and MDA levels (C) were measured in I/R-treated mice with leucine supplementation. (D-F) Serum levels of ALT (D), AST (E) and LDH (F) were measured in I/R-treated mice with leucine supplementation. (G) Representative H&E-stained liver sections from indicated mice. Significance was calculated by Student's t-test; *P<0.05, **P<0.01

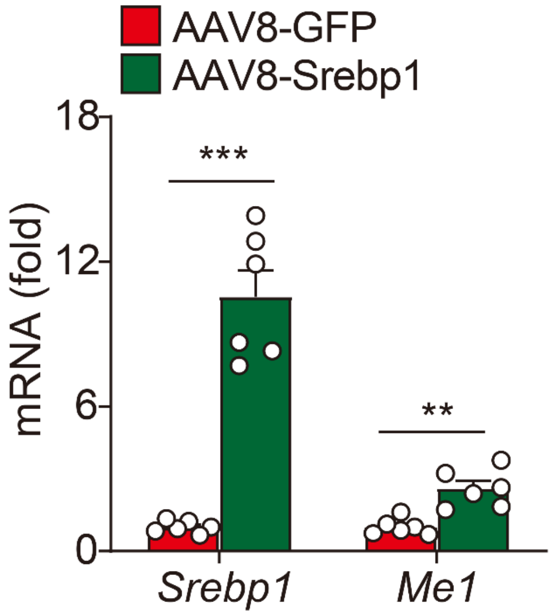

Figure S11. Hepatic mRNA of *Srebp1* and *Me1* were measured in mice treated with AAV8-Srebp1 or AAV8-GFP. Significance was calculated by Student's *t*-test; *P<0.05, **P<0.01, ***P<0.001.

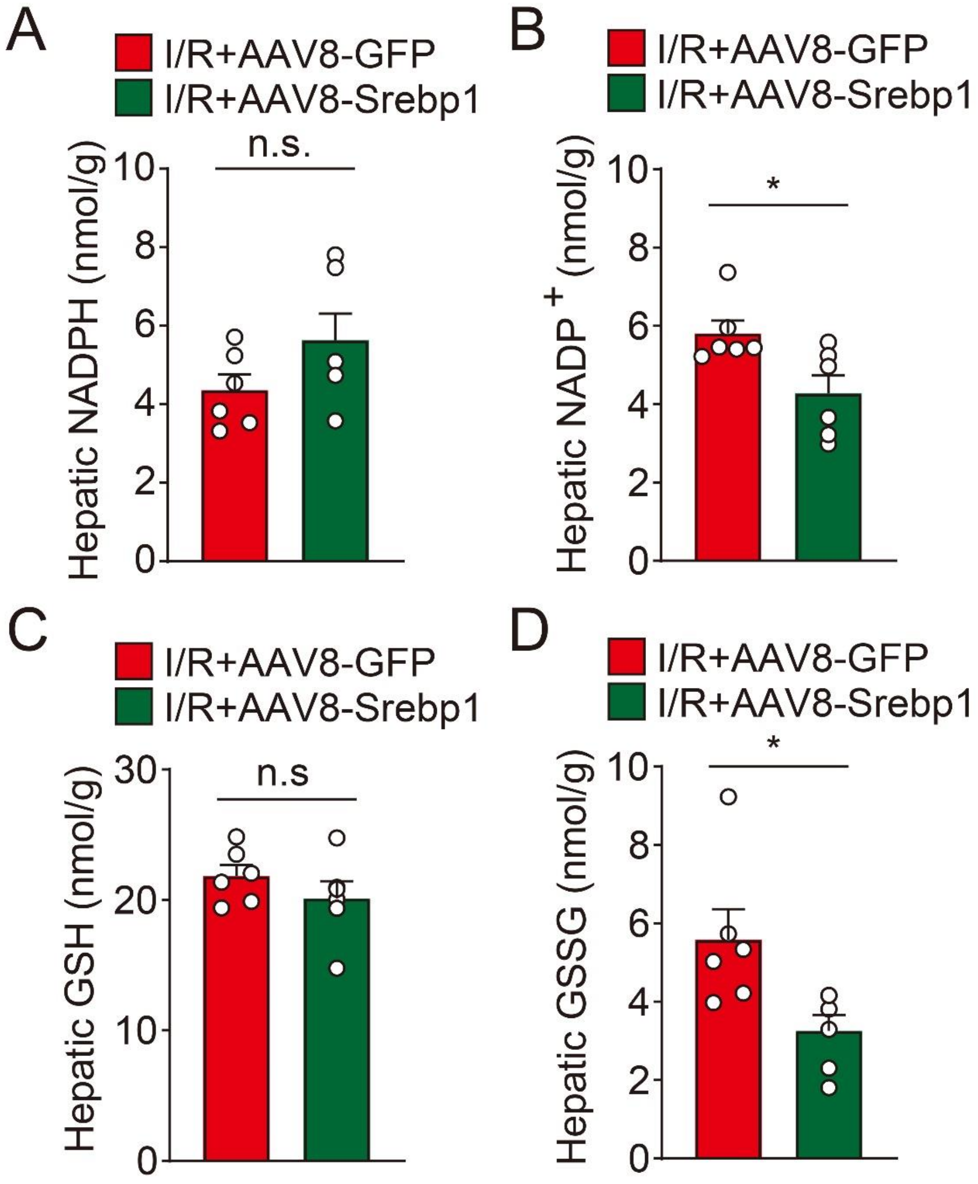

Figure S12. Hepatic levels of NADPH (A), $NADP^+$ (B), GSH (C), and GSSG (D) were measured in I/R-treated mice with or without AAV-mediated *Srebp1* overexpression. Significance was calculated by Student's *t*-test; *P<0.05, n.s.=not significant.

Table S1. Primers used for genotyping and real-time PCR analysis.

Gene	Primer sequence (5'→3')
**Genotyping**	
Me1 forward	TAGCCGCACGCTGATGATAG
Me1 reverse	GCAGCTGTCAGACTAGCCAA
Alb-Cre common	TGGCAAACATACGCAAGGG
Alb-Cre mutant	CGGCAAACGGACAGAAGCA
Alb-Cre mutant	GGCAATGGTTCCTCTCTGCT
**Real-time PCR**	
Gapdh forward	ATCATCCCTGCATCCACT
Gapdh reverse	ATCCACGACGGACACATT
Ptgs2 forward	CTGCGCCTTTTCAAGGATGG
Ptgs2 reverse	GGGGATACACCTCTCCACCA
Me1 forward	GTCGTGCATCTCTCACAGAAG
Me1 reverse	TGAGGGCAGTTGGTTTTATCTTT
Me2 forward	TACCACTCCTTGTACCTTGACC
Me2 reverse	TCTTGTAACGTAAACGCCATTCC
G6pd forward	TCAGACAGGCTTTAACCGCAT
G6pd reverse	CCATTCCAGATAGGGCCAAAGA
6pgd forward	TACAGACACGAGATGCTGCC
6pgd reverse	TGAGCCCCAAAGTAATCCCG
Idh1 forward	GGTTATGGCTCCCTTGGCAT
Idh1 reverse	CCCTTTCTGGTACATGCGGT
Idh2 forward	GGAGAAGCCGGTAGTGGAGAT
Idh2 reverse	GGTCTGGTCACGGTTTGGAA
Mthfd1 forward	GGGAATCCTGAACGGGAAACT
Mthfd1 reverse	TGAGTGGCTTTGATCCCAATC
Mthfd2 forward	AGTGCGAAATGAAGCCGTTG
Mthfd2 reverse	GACTGGCGGGATTGTCACC

Aldh1l1 forward	CAGGAGGTTTACTGCCAGCTA
Aldh1l1 reverse	CACGTTGAGTTCTGCACCCA
Aldh1l2 forward	ACCAGCCGGGTTTATTTCAAA
Aldh1l2 reverse	ACTCCCACTACTCGGTGGC

Table S2. Antibodies used in this study.

Antibody	Application	Description	Source	Catalog no.
**Primary Antibodies**				
anti-Me1	WB/IHC	Rabbit Polyclonal	Proteintech	16619-1-AP
anti-Pten	WB	Rabbit Monoclonal	Cell Signaling	9559
anti-mTOR	WB	Rabbit Monoclonal	Cell Signaling	2983
anti-P-mTOR	WB	Rabbit Monoclonal	Cell Signaling	5536
anti-Akt	WB	Rabbit Monoclonal	Cell Signaling	4691
anti-P-Akt	WB	Rabbit Monoclonal	Cell Signaling	4060
anti-S6K1	WB	Rabbit Monoclonal	Cell Signaling	2708
anti-P-S6K1	WB	Rabbit Polyclonal	Cell Signaling	9208
anti-Srebp1	WB/ChIP	Mouse Monoclonal	Santa Cruz	sc-13551
anti-Gapdh	WB	Mouse Monoclonal	Proteintech	60004-1-Ig
**Secondary Antibodies**				
anti-mouse IgG	WB	Goat	Proteintech	SA00001-1
anti-rabbit IgG	WB	Goat	Proteintech	SA00001-2

IHC, immunohistochemistry; WB, western blotting.
